# The Effect of a Unilateral Orchiectomy before Gonadotoxic Treatment on the Contralateral Testis in Adult and Prepubertal Rats

**DOI:** 10.1371/journal.pone.0164922

**Published:** 2016-10-21

**Authors:** Charlotte Rombaut, Katrien Faes, Ellen Goossens

**Affiliations:** Biology of the Testis, Research Laboratory for Reproduction, Genetics and Regenerative Medicine, Vrije Universiteit Brussel (VUB), Brussels, Belgium; University Hospital of Münster, GERMANY

## Abstract

**Purpose:**

Previous studies have shown that the removal of one testis leads to a compensatory mechanism in the contralateral one, but this was species and age dependent. The aim of this study was to check whether this compensation would still occur after the combination of a unilateral orchiectomy and gonadotoxic treatment, since this resembles the clinical situation of patients who have to undergo highly toxic cancer treatment and therefore choose to cryopreserve a testicular biopsy for fertility restoration purposes.

**Materials & Methods:**

Sprague Dawley rats underwent either unilateral orchiectomy, gonadotoxic busulfan treatment, the combination of both or served as fertile control. A comparison of the compensatory effects was made between adult and prepubertal treated rats. Mating experiments were performed, testosterone levels were followed-up, testicular weight was recorded and histology was analysed.

**Results:**

Adult treated rats were able to restore fertility spontaneously in all treatment groups. On the other hand, 30% of the rats that underwent a unilateral orchiectomy and gonadotoxic treatment at prepubertal age showed hampered spermatogenesis, low testosterone levels, decreased testicular weights and were not able to reproduce.

**Conclusion:**

This study emphasizes the need of fertility preservation strategies in prepubertal patients before gonadotoxic interventions.

## Introduction

Cancer occurs in one out of 600 children but because of the improved treatments, 80% survive this disease (Belgian Cancer Registry, www.kankerregister.org, Accessed August 2, 2016). Unfortunately, the highly sensitive prepubertal testicular tissue might be damaged due to the chemo- or radiotherapy, leading to destruction of the spermatogonial stem cells (SSCs) [1]. If all SSCs are destroyed during cancer therapy, patients become infertile [2,3]. Adult men are able to cryopreserve their semen before onset of the cancer treatment in order to retain their fertility by the use of assisted reproductive technology (ART). On the contrary, prepubertal boys are lacking spermatogenesis and therefore other fertility preservation methods need to be developed depending on the underlying cause of the SSC loss [4,5,6]. In that prospect, a testicular biopsy is taken to cryopreserve SSCs. Since (partial) orchiectomy is performed in order to bank prepubertal tissue for later restoration of spermatogenesis, the remaining testis might work compensatory. Different research groups have investigated the potential of the remaining testis to upregulate spermatogenesis after a unilateral orchiectomy but the outcome of the studies differed. Noller et al. showed that after a unilateral orchiectomy in adult guinea pigs the amount of deoxyribonucleic acid (DNA) and ribonucleic acid (RNA) was raised in the contralateral testis [7]. The ratio of RNA to DNA was not affected which suggested that the compensatory growth was due to hyperplasia and not hypertrophy. Another research group investigated the morphologic response of Leydig cells of adult rats to hemicastration. No compensatory testicular hyperthrophy was noted. However, the interstitium was enlarged. Leydig cell hypertrophy and hyperplasia and a compensatory testicular hypersecretion of testosterone were observed. This study suggests that hemicastration does not lead to changes in the hypothalamo-hypophyseal axis but that it leads to a compensatory hyperactivity and hypersecretion of the Leydig cells [8]. Subsequently, the effect of a unilateral orchiectomy after spermatic cord torsion was evaluated in adult rats. No significant differences in serum testosterone levels were found and the nuclei of the Leydig cells in the contralateral testis were significantly enlarged. As the volume of the remaining testis was also increased by 21% compared to the control groups, it can be suspected that orchiectomy could lead to a compensatory hypertrophy of the contralateral testis resulting in the production of as much testosterone as two testes of intact rats [9].

Seven months after performing a unilateral orchiectomy on neonatal and prepubertal rabbits, no increase was observed in testis weight, Sertoli cell number, sperm production or sperm output in either age group. Compared to the results in adult models, this means that there might be a compensatory mechanism after a unilateral orchiectomy when the surgery is performed on sexually mature animals but not in prepubertal animals [10]. Contradictory, another study found that prepubertal hemicastration in rabbits led to compensatory hypertrophy in the contralateral testis when puberty was reached [11].

A study about a unilateral orchiectomy in combination with cancer treatment was performed on rats to investigate the role of peritubular myoid cells in postnatal testicular growth. In order to downregulate proliferation of peritubular myoid cells, the tyrosine kinase inhibitor, Imatinib mesylate was used [12]. An increased length of the seminiferous cords and testicular weight was observed after a unilateral orchiectomy. These effects were blocked if the unilateral orchiectomy was combined with Imatinib mesylate [13]. No difference on the number of germ cells was detected if a unilateral orchiectomy or the combined treatment was induced. However, the reproductive capability of the rats treated with the combination of a unilateral orchiectomy and cancer treatment was not investigated. Also, animal data suggest that this tyrosin kinase inhibitor does not impair fertility meaning that this model is not representative for the boys in need of fertility preservation [14].

Therefore, it is necessary to investigate the effect of a unilateral orchiectomy combined with gonadotoxic treatment, like the alkylating agent busulfan, to mimic the clinical situation. If it appears that testicular compensation due to the unilateral orchiectomy can override the harmful effects of the gonadotoxic treatment and lead to spontaneous fertility restoration, no further invasive fertility restoration techniques need to be applied. If proven otherwise, more research towards fertility restoration needs to be performed in order to give these patients a chance of fathering their own children.

## Materials & Methods

### Animal model

**Fig 1:**
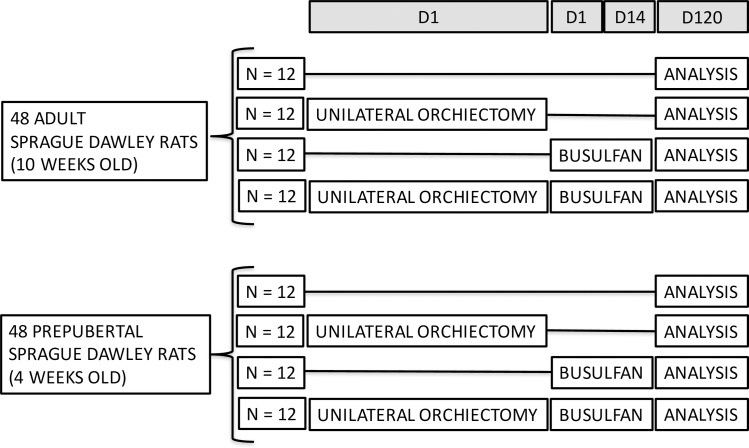
Experimental design. A total of 48 adult and 48 prepubertal Sprague Dawley rats were divided into four treatment groups. The first group served as a fertile control. The second group underwent unilateral orchiectomy. The third group was treated with busulfan injections. The last group got the combination of a unilateral orchiectomy and busulfan injections. D: day.

Male Sprague Dawley rats (Charles River Laboratories, Leiden, Netherlands) (total N = 96) were assigned to one of the four experimental groups: rats serving as a fertile control, undergoing unilateral orchiectomy, busulfan treatment or the combination of unilateral orchiectomy and busulfan treatment. One experiment was conducted with 12 adult (10 weeks old) or 12 prepubertal (4 weeks old) rats per group (Fig 1).

### Unilateral orchiectomy and busulfan injection

Random assignment was done to determine which testis to remove in the rats undergoing unilateral orchiectomy. The animals were sedated via an intraperitoneal injection (IP; 125μl/100 g bodyweight) with a mixture of 0.33mg/ml medetor (83255102; Virbac, Leuven, Belgium) and 50mg/ml ketamine (6905306; Ceva Santé Animale, Machelen, Belgium). In adult rats, an incision of 1 cm was made in the scrotum, while in prepubertal rats the incision was made in the abdomen and peritoneum. A double ligature of digestible vicrylTM Plus thread (VCP303H; Ethicon, Norderstedt, Germany) was placed around the vas deferens and the blood supply before removing the testicle. After removal of the testis, the scrotum or abdomen and peritoneum were closed using Catgut absorbable surgical sutures (2151512; SMI, St Vith, Belgium). Subcutaneous injections of 2.5% Baytril (0.1ml; 79828047; Bayer, Antwerpen, Belgium) were given to avoid post-operative infections. Busulfan was administered IP in two doses of 10mg/kg bodyweight (154906; ICN Biomedicals inc, Irvine, USA) with a time interval of fourteen days [15].

### Blood sampling and ELISA

At day 1, 14, 60 and 120 of the experiment, blood samples were taken through the tail vein [16]. Serum was collected and stored at -80°C. Serum testosterone levels were measured by a competitive ELISA kit according to manufacturer’s instructions (ab108666; Abcam, Cambridge, UK).

### Mating

One day after the surgery, every adult male was placed with two adult female Sprague Dawley rats during four gestation periods. Every gestation period counted 23 days. Since the prepubertal males were not sexually mature at the time of the surgery, they were mated at the age of ten weeks, also for four gestation periods.

### Euthanasia and collection of the remaining testis

Four months after the surgery, all animals were killed with an overdose (60mg/ml IP) of Nembutal. The total bodyweight was recorded. An incision was made in the scrotum and the remaining testis was collected in Dulbecco’s modified Eagle medium (31331; Life Technologies, Gent, Belgium). For the animals that did not undergo orchiectomy, the right or left testis was randomly collected. Straight after collection of the testis, the tunica albuginea was removed. The decapsulated weight and the total body weight of the rats were analysed.

### Histology

Four 5 μm-thick sections differing by 50 μm were made for each staining protocol. Ten round tubules per depth (with a ratio of less than 1.5 between the longest diameter of the tubule and the diameter perpendicular to it [17]) were analyzed per rat.

To evaluate the level of spermatogenesis, a standard hematoxylin and eosin (HE) staining was performed. The slides were visualized with an Olympus IX 81 inverted microscope (Olympus U- TB190, Tokyo, Japan) at a magnification of x10.

To test whether busulfan, a unilateral orchiectomy or the combination of both had an effect on the number of spermatogonia per tubule, a ubiquitin carboxy-terminal hydrolase L1 (UCHL1) staining was performed. The UCHL1 protein, also referred to as protein gene product 9.5 (PGP9.5), is expressed in the cytoplasm of undifferentiated type A spermatogonia. After deparaffinization with xylene, sections were dehydrated by successive series of ethanol (100%, 100%, 90% and 70%). After washing in phosphate buffered saline (PBS; 70011036; Life Technologies), antigen retrieval was performed by a 75-minute water bath treatment using citric acid (pH 6). After another washing step in PBS, the sections were blocked for non-specific binding with Casblock (008120; Life Technologies) during 10 minutes. The first antibody, anti-UCHL1 (1:750 dilution; rabbit anti-human PGP9.5; A5978630504; Bio- Connect Diagnostics, Huissen, The Netherlands) was added and the sections were incubated in a humidified chamber for two hours at 4°C. After three washing steps in PBS, the slides were incubated with donkey anti-rabbit Alexa-Fluor 488 (1:200 dilution; A21206; Life Technologies, Merelbeke, Belgium) conjugated secondary antibody for 1 hour at RT. After washing 3 x 5 min in PBS the slides were mounted with 4’,6 diamidino-2-phenylindole (DAPI; S36939; Invitrogen, Merelbeke, Belgium) and imaged on an Olympus IX 81 inverted microscope using 405 nm (DAPI) and 488 nm (UCHL1) filters. Isotype controls were taken along.

### Statistics

Statistical significance in cell counts, mating results and testis weight was determined by a one-way Anova and Tukey post-hoc test (Graphpad Prism Version 5.0b, San Diego, USA). A repeated measures Anova was performed to determine statistical significance in testosterone levels (IBM SPSS Statistics Version 22, IBM Corporation, Somers, NY, USA). Results are shown as mean ± standard deviation. A p-value of less than 0.05 was considered statistically significant.

### Ethical approval

The ethical committee for the use of lab animals at the Vrije Universiteit Brussel has approved all handlings in this experiment (project number 14-216-2)

## Results

### The combination of a unilateral orchiectomy and busulfan injections compromises serum testosterone levels if treatment was induced at prepubertal age

**Fig 2:**
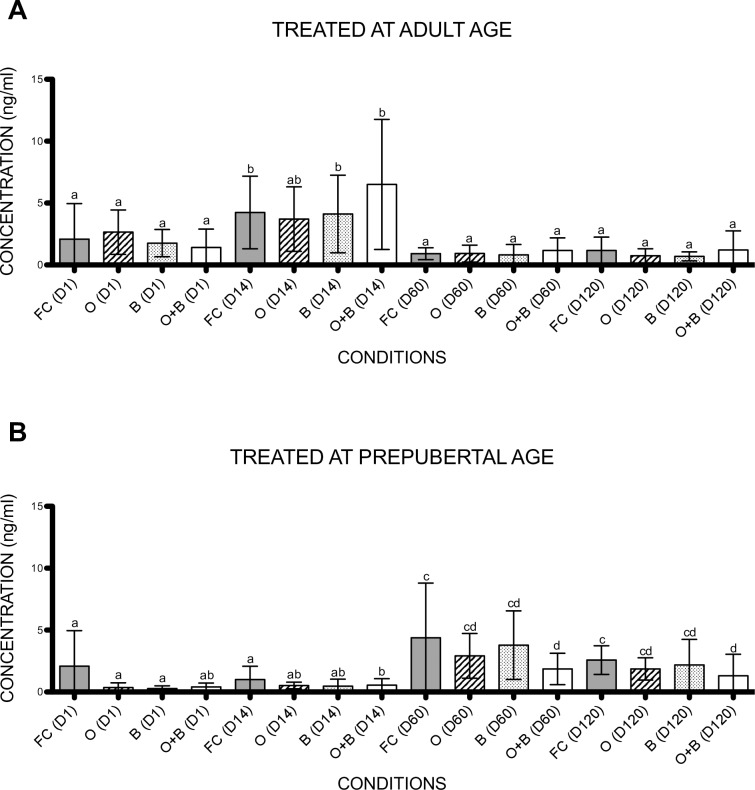
Testosterone levels. Evolution of testosterone levels of rats treated at adult (a) or prepubertal (b) age. Variables with a different letter are statistically different. FC: fertile controls, O: rats treated with unilateral orchiectomy, B: rats treated with busulfan, O+B: rats treated with busulfan and unilateral orchiectomy, D: day.

At day 1, 14, 60 and 120 after treatment, testosterone levels were determined by ELISA. First, testosterone levels were compared between time points for each of the different experimental groups. Concerning the adult rats, at day 1, no significant differences were seen between the treatment groups, meaning that all rats started off with the same level of testosterone before any treatment was executed. An increase in testosterone level was noted at day 14 compared to day 1 for the fertile control group (p = 0.034), the busulfan treated group (p = 0.015) and the combined treatment group (p = 0.002) most likely due to the hormonal boost caused by the mating experiment [18]. When day 60 was compared to day 14, a significant decrease of testosterone level was noted in all four experimental groups (fertile control p = 0.001, unilateral orchiectomy p = 0.002, busulfan p = 0.001, combined treatment p = 0.005). No significant differences were noted between day 60 and 120. Second, a comparison of testosterone levels was made between the different experimental groups. No significant differences were found (Fig 2A) and therefore, all adult experimental groups were able to produce the same amounts of testosterone during the full duration of the experiment.

For the prepubertal treated groups, no significant differences were obtained between day 1 and 14. At day 60 of the experiment, a significant increase in testosterone level was observed for all the treatment groups (fertile control p = 0.015, unilateral orchiectomy p = 0.001, busulfan p = 0.001, unilateral orchiectomy combined with busulfan p = 0.003) which was most likely due to the hormonal boost caused by pubertal development and mating [18]. These levels were stable till the end of the experiment (day 120). Next, a comparison of testosterone levels was made between the different prepubertal experimental groups. Although the experimental groups that only underwent a unilateral orchiectomy or those that only received gonadotoxic treatment were able to produce a similar amount of testosterone compared to the fertile controls, the combination of both interventions hampered hormone production leading to a significant decrease in testosterone production at day 120 (p = 0.024) compared to the fertile controls (Fig 2B).

### Testicular weight is lower after combined treatment

**Fig 3:**
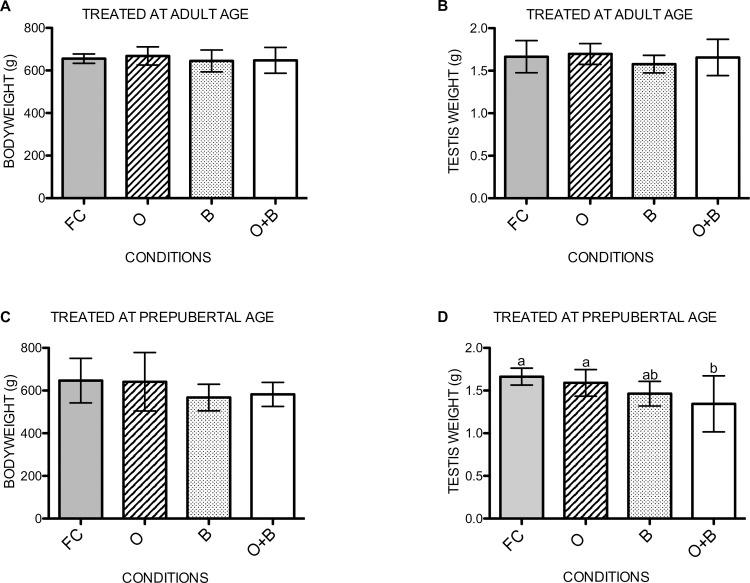
Testis weight. Bodyweight (A) and testicular weight (B) of the adult treated rats at the time of euthanasia. Bodyweight (C) and testicular weight (D) of the prepubertal treated rats at the time of euthanasia. Variables with a different letter are statistically different. If no letters are indicated, no statistical differences were found in the graph. FC: fertile controls, O: rats treated with unilateral orchiectomy, B: rats treated with busulfan, O+B: rats treated with busulfan and unilateral orchiectomy.

Four months after treatment, the testicular weight and the total body weight of the rats was determined. No significant differences in body weight were noted in both age categories (Fig 3A and 3C). The adult treated groups showed no differences in testicular weight after four months (Fig 3B). On the other hand, the testis weight of the rats that underwent a unilateral orchiectomy and busulfan treatment at prepubertal age showed a significant lower testis weight compared with those who only underwent a unilateral orchiectomy (p = 0.031) or served as a fertile control (p = 0.004) (Fig 3D).

### The combined treatment negatively hampers spermatogenesis in rats treated at prepubertal age

**Fig 4:**
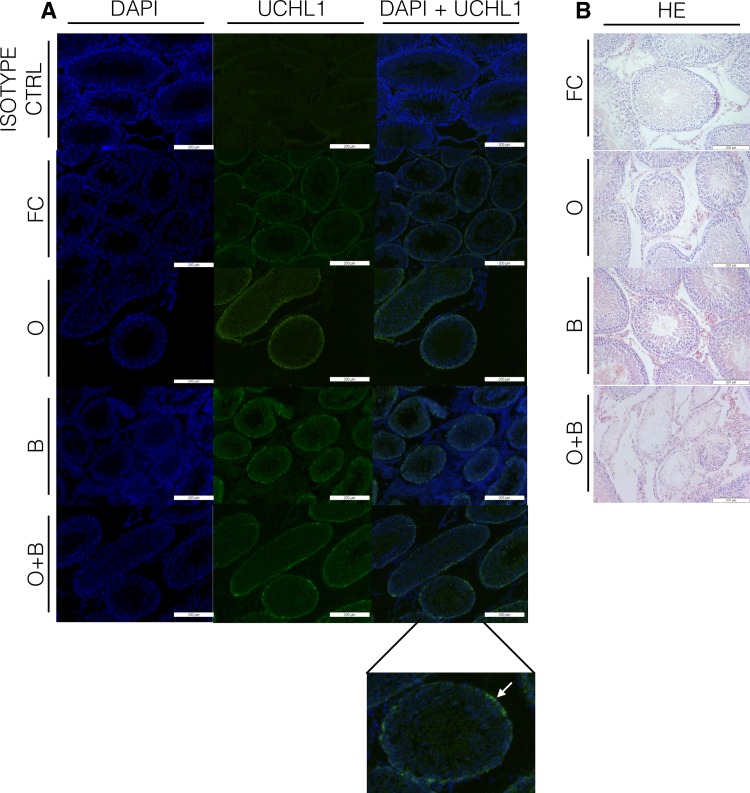
Evaluation of spermatogenesis and number of spermatogonia. a) A UCHL1 staining was performed to evaluate the number of spermatogonia (white arrow) per tubule. All pictures show testis sections of prepubertal treated rats. Left row: DAPI, middle row: UCHL1, right row: merge UCHL1+DAPI. b) A HE staining was performed to evaluate spermatogenesis. All pictures show testis sections of prepubertal treated rats. Thirty percent of the rats that received the combined treatment at prepubertal age showed SCO tubules. FC: fertile controls, O: rats treated with unilateral orchiectomy, B: rats treated with busulfan, O+B: rats treated with busulfan and unilateral orchiectomy.

**Fig 5:**
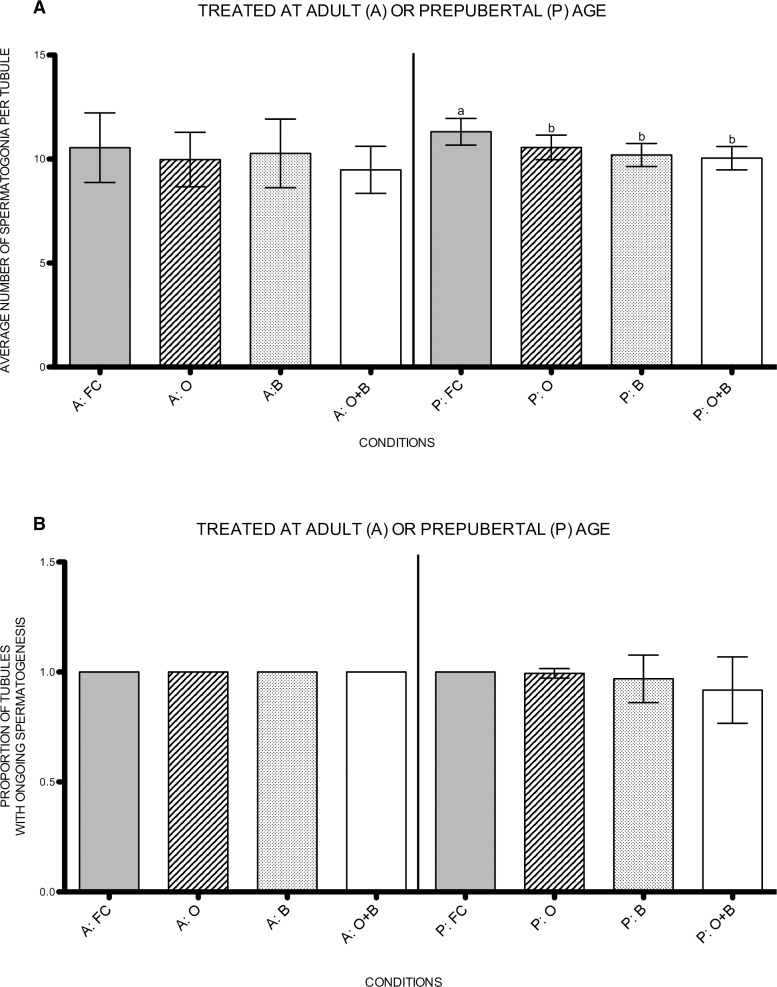
Histology. a) Average number of spermatogonia per tubule. Variables with a different letter are statistically different. If no letters are indicated, no statistical differences were found in the graph. b) Proportion of tubules with ongoing spermatogenesis. A: treated at adult age, P: treated at prepubertal age, FC: fertile controls, O: rats treated with unilateral orchiectomy, B: rats treated with busulfan, O+B: rats treated with busulfan and unilateral orchiectomy.

To determine the effect of the different treatments on the pool of spermatogonia, a UCHL1 staining was performed (Fig 4A). In the rats treated at adult age, none of the treatments had a significant impact on the number of spermatogonia per tubule (Fig 5A).

A significant decrease in number of spermatogonia per tubule was seen in the rats that underwent a unilateral orchiectomy (p = 0.012), busulfan treatment (p = 0.001) or the combination of both (p = 0.001) at prepubertal age (Fig 5A).

A HE staining was performed to evaluate spermatogenic recovery (adult) or initiation (prepubertal) of spermatogenesis (Fig 4B). All adult animals were able to restore spermatogenesis, irrespective the treatment (Fig 5B). However, one rat that underwent a unilateral orchiectomy and one rat that received busulfan at prepubertal age showed a few tubules lacking spermatogenesis (respectively 8% and 37%) next to tubules with ongoing spermatogenesis. However, no significant differences were reached compared to the fertile controls. In the rats that underwent the combined treatment at prepubertal age, 30% of the rats showed Sertoli-cell-only (SCO) tubules (28% ± 0.460) next to tubules with ongoing spermatogenesis (Fig 5B).

### Offspring production is hampered in rats receiving the combined treatment at prepubertal age

Mating experiments were performed between one male and two females to determine the potential to recover fertility after the different treatments. After 4 gestation periods, the number of litters would be 8 per male rat if all females produced a litter every gestation period. Also, the mean number of pups per litter was analyzed.

**Fig 6:**
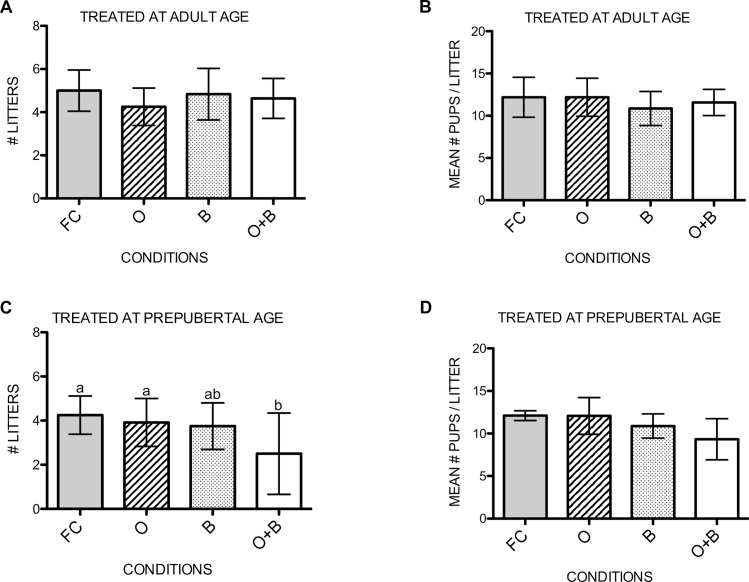
Mating. Number of litters produced during the whole mating experiment per adult (a) or prepubertal (c) treated rat per gestation period during 4 months of mating. Mean number of pups per litter produced per adult (b) or prepubertal (d) treated rat per gestation period. Variables with a different letter are statistically different. If no letters are indicated, no statistical differences were found in the graph. FC: fertile controls, O: rats treated with unilateral orchiectomy, B: rats treated with busulfan, O+B: rats treated with busulfan and unilateral orchiectomy.

The number of litters produced by the adult treated animals did not differ between the experimental groups (Fig 6A). Subsequently, no significant differences were noted in the number of pups per litter for all experimental groups (Fig 6B).

On the other hand, if rats underwent a unilateral orchiectomy and busulfan treatment at prepubertal age, the number of litters produced during the four gestation periods was significantly lower than those produced by the fertile control group (p = 0.008) and the group that underwent a unilateral orchiectomy (p = 0.036) (Fig 6C). Thereby, 30% of the rats of this combined treatment group were not able to produce any offspring during the complete mating experiment. But, if litters were produced, no significant differences were noted in the number of pups per litter for all experimental groups (Fig 6D).

In both age categories, the ratio of female/male pups was normal (ratio 48/52).

## Discussion

Gonadotoxic treatment can cause permanent infertility due to the deleterious effects on the rapidly dividing stem cell pool [5]. Adult men have the possibility to cryopreserve their semen before gonadotoxic treatment in contrast to prepubertal boys who lack full spermatogenesis. New preservation techniques are being developed for these prepubertal boys who need to undergo gonadotoxic treatment. As part of any fertility preservation strategy, a large testicular biopsy is taken.

Several reports using different animal models have been published on the effect of unilateral orchiectomy. Although a compensatory effect after a unilateral orchiectomy has been described in adult models [7–9, 19–22], no consensus was reached for prepubertal animals [10,11]. Given the reports in literature on the increase of SSC self-renewal after gonadotoxic treatment [5,23] and compensatory mechanisms in the remaining testis after a unilateral orchiectomy, it might be possible that such compensatory mechanism also occurs after a unilateral orchiectomy followed by gonadotoxic treatment. This hypothesis has never been addressed with highly gonadotoxic agents. However, for clinical application it is important to know whether invasive fertility restoration therapies (like SSC transplantation or intratesticular grafting) are necessary or whether it is possible that fertility restores spontaneously through compensation.

The occurrence of a compensatory mechanism after unilateral orchiectomy has been related to an increase in testicular weight [7,8,19,21]. The testicular weight is an exquisitely sensitive marker of sperm production [24]. However, no increase in testicular weight was noted in the prepubertal and adult group treated with a unilateral orchiectomy, possibly because of different compensatory mechanisms in different species (guinea pig [7], rabbit [10]), a short-term follow up (15 days) [8] or variations in the experimental set-up (spermatid cord torsion prior to orchiectomy) [9]. On the other hand, a significantly lower testicular weight was observed in the group treated with a unilateral orchiectomy and busulfan at prepubertal age suggesting a deficit in sperm production.

The measurement of testosterone levels showed that rats that underwent a unilateral orchiectomy at adult or prepubertal age could compensate and produce the same hormone levels as the rats with two intact testes. Also, in both age categories, busulfan treatment did not hamper testosterone production. However, although this was not the case for the adult treated rats, significantly lower concentrations were found in the experimental group that underwent a unilateral orchiectomy and busulfan treatment at prepubertal age showing that the combined treatment could not induce a mechanism to compensate for testosterone loss. In a future study, it would be interesting to also measure LH and FSH levels to get a broader perspective of the gonadotoxic effects on steroidogenesis.

A UCHL1 immunostaining was performed to determine the effect of the different treatments on the spermatogonial pool. In all adult treated rats, no significant differences in spermatogonial count were observed which could be explained by the activation of the quiescent SSCs to repopulate the testis after gonadotoxic treatment [23,25,26].

In the prepubertal treated animals, a significant lower amount of UCHL1-positive cells was seen in all treatments groups compared to the fertile controls. Since prepubertal animals feature a highly proliferative SSC pool, these cells are more susceptible to alkylating agents like busulfan [27]. Nevertheless, testes with complete depletion of spermatogonia were never found meaning that in every experimental group, at least some SSCs survived the treatment and were able to proliferate and differentiate.

HE stainings were performed to evaluate the spermatogenic recovery (adult) or initiation of spermatogonial differentiation (prepubertal). All adult treated experimental groups demonstrated a full spermatogenic recovery, whereas 30% of the rats that underwent a unilateral orchiectomy and gonadotoxic treatment at prepubertal age could not restore spermatogenesis. Also, one rat that received busulfan and one rat that underwent a unilateral orchiectomy at prepubertal age showed a few SCO tubules. Therefore, although busulfan treatment or a unilateral orchiectomy demonstrated a minor impact on spermatogonial differentiation, the combination of both led to a significant disturbance in spermatogenesis. Nevertheless, none of the treatments induced a complete depletion of spermatogenesis.

In the last part of this study, fertility restoration was evaluated by mating experiments. In adult rats, no differences in mean number of litters were found compared to the fertile controls, irrespective of the treatment. Therefore, rats with only one testis were able to produce as many litters as those with two intact testes.

Both unilateral orchiectomy and gonadotoxic treatment did not affect fertility if applied separately at prepubertal age. If combined, a significant decrease in number of litters was reached. Noteworthy, 30% of the rats were not able to produce any offspring during the whole mating experiment. Interestingly, these infertile rats also showed lower testicular weight, lower testosterone levels, less spermatogonia per tubule and presented SCO tubules. On the other hand, none of these sterile rats showed a complete depletion of spermatogenesis suggesting other underlying problems. Therefore, in a future study, it would be interesting to also examine sperm parameters like sperm count, motility and morphology. We can thus conclude that, in prepubertal rats, unilateral orchiectomy can induce a compensatory mechanism, but not when the remaining testis has been damaged by gonadotoxic treatment. However, the number of pups per litter was not affected.

Although, in this experiment, no obvious physical abnormalities were observed in the pups, persistent biological effects caused by the gonadotoxic treatment can have a transgenerational impact. In adult models, these effects are well known leading to reduced fertility in the offspring [28,29]. In prepubertal animals, transgenerational gonadotoxic effects have not been studied yet.

Since gonadotoxic treatment alone led to compensation in both age groups, the question now rises whether avoiding a biopsy and hoping for compensation in time is a better alternative. However, the effects of gonadotoxic agents are species-dependent. In rats, the administered dose (2 times 10mg/kg) to destroy endogenous spermatogenesis is a lot milder than the dose used in mice (1 time 40mg/kg) since they are more susceptible to the deleterious systemic effects of gonadotoxic treatment and higher doses can be lethal. Although this rat model resembles the clinical situation where there is a high risk of losing fertility after gonadotoxic treatment, the milder dose may have lead to higher chances of spermatogonial survival and spontaneous recovery of spermatogenesis. In the clinic, children and men treated with gonadotoxic agents have a high risk of permanent sterility [30]. The chance of spermatogenic recovery after chemotherapy depends on the received dose and the used gonadotoxic agent [5]. Since chemotherapy kills cells as they divide, the SSCs of children are more susceptible to the therapy than the ones from adults because of their higher mitotic rate [3]. For radiotherapy, recovery depends on the received dose, the scattered radiation and the fractionation of the radiation bundles. Next to the higher susceptibility of the prepubertal stem cell pool, the shorter trunk length causes a greater risk for scattered radiation in children compared to larger (adult) individuals [2]. Hence, by not taking a biopsy in prepubertal boys before gonadotoxic treatment, the possibility for fertility restoration can fall through. As differences in compensation occur between rats and humans, we could advise to repeat this study using an animal model with closer anatomical and endocrinological similarities to humans like non-human primates. Studies in non-human primates confirmed the more detrimental effects of gonadotoxic treatments on the prepubertal testis compared to pubertal or adult models [31,32].

Studies concerning a unilateral orchiectomy in prepubertal boys are nonexistent and those in adult men are limited and divergent. The effect on the endocrine parameters and the semen quality of patients who underwent orchiectomy after unilateral testicular injury was studied. Normal sperm density was found next to normal sperm motility. Increased levels of FSH and LH were seen after the orchiectomy that could point out a stimulation of spermatogenesis. Petersen et al. looked at the semen quality and serum hormones (LH, FSH, testosterone) 48 hours before and 48 hours after orchiectomy in men with testicular cancer who did not underwent any gonadotoxic treatment yet [22]. Here, median sperm concentration and total sperm counts were decreased after orchiectomy whereas FSH levels were increased. Next, the long-term effect on spermatogenesis and the exocrine function after unilateral orchiectomy was studied in adult patients with testicular cancer. One year after the orchiectomy, nine patients went from oligospermic to normospermic state with increased sperm counts. However, patients with raised FSH levels from the start were not able to recover spermatogenesis, implying that disturbances of spermatogenesis are mostly permanent when baseline azoospermia or oligospermia is combined with high levels of FSH. Fifty percent of the observed men were able to father children, mostly by the help of ART [20].

In conclusion, rats that underwent a unilateral orchiectomy and busulfan injections at adult age could compensate and restore fertility. If the combined treatment was executed in rats at prepubertal age, disturbed compensation provoked sterility. Therefore, this study should be repeated in animals like non-human primates in order to understand whether prepubertal boys, who have banked testicular tissue before gonadotoxic therapy, are indeed in need of restoration strategies like SSCT [33] and intratesticular grafting [34].
